# Oral and oropharyngeal cancer: epidemiology and survival analysis

**DOI:** 10.1590/S1679-45082018AO4248

**Published:** 2018-05-29

**Authors:** Juliana da Silva Moro, Marília Cunha Maroneze, Thiago Machado Ardenghi, Luisa Machado Barin, Cristiane Cademartori Danesi

**Affiliations:** 1 Universidade Federal de Santa Maria, Santa Maria, RS, Brazil

**Keywords:** Mouth neoplasms/epidemiology, Survival rate, Oropharyngeal neoplasms/epidemiology, Prognosis, Neoplasias bucais/epidemiologia, Taxa de sobrevida, Neoplasias orofaríngeas/epidemiologia, Prognóstico

## Abstract

**Objective:**

To evaluate the epidemiological profile and survival rate of oral and oropharyngeal cancer patients seen at a university hospital.

**Methods:**

A cross-sectional study was carried out by means of the pathological reports of patients with oral and oropharyngeal cancer, seen at a university hospital of the Southern Region, between January 2004 and December 2014. Information was collected on patients and tumors. The mortality rate was gathered from the patient death registry in the Mortality Information System. Data were analyzed using the Kaplan-Meier survival curve and the log-rank test to compare variables.

**Results:**

The 5- and 10-year survival rates were 42% and 38%, respectively. The anatomical location had a significant association with survival rate (p=0.001), with the rates were better in the lips (p=0.04), and worse in the oropharynx (p=0.03). There were no statistically significant differences between survival rates according to age, sex, ethnicity, schooling level and histologic grade.

**Conclusion:**

The survival rates of oral and oropharyngeal cancer were and associated with the anatomical site of the tumor.

## RESUMO

**Objetivo:**

Avaliar o perfil epidemiológico e a taxa de sobrevida do câncer de boca e orofaringe de pacientes atendidos em um hospital universitário.

**Métodos:**

Foi realizado um estudo transversal por meio dos laudos anatomopatológicos dos pacientes com câncer de boca e orofaringe atendidos em um hospital universitário, na Região Sul, entre janeiro de 2004 a dezembro de 2014. A partir destes laudos, foram coletadas informações sobre o paciente e o tumor. A taxa de mortalidade foi obtida do registro de óbitos dos pacientes no Sistema de Informações sobre Mortalidade. Os dados foram analisados utilizando a curva de sobrevida pelo método de Kaplan-Meier e o teste de log-rank para a comparação das variáveis.

**Resultados:**

As taxas de sobrevida em 5 e 10 anos foram 42% e 38%, respectivamente. A localização anatômica apresentou associação significativa com a taxa de sobrevida (p=0,001), sendo que, em lábio, os índices foram melhores (p=0,04) e, em orofaringe, piores (p=0,03). Não houve diferenças estatisticamente significantes entre as taxas de sobrevida de acordo com idade, sexo, etnia, nível educacional e grau histológico.

**Conclusão:**

As taxas de sobrevida do câncer de boca e orofaringe foram baixas e associadas à localização anatômica do tumor.

## INTRODUCTION

Head and neck cancers are a serious public health problem due to their high incidence, prevalence, and mortality.^(^ ^1^ ^)^ Tumors of the mouth and oropharynx are some of the most frequent in this group,^(^ ^2^ ^)^ and accounted for more than 219 thousand deaths worldwide, in 2012.^(^ ^3^ ^)^ Approximately 90% of them are squamous cell carcinoma (SCC).^(^ ^4^ ^)^

The majority of patients diagnosed with oral and oropharyngeal cancer have a history of smoking and alcohol consumption, which are important etiologic factors.^(^ ^5^ ^)^ Further, human papillomavirus (HPV) infection has been associated with development of oropharyngeal cancer.^(^ ^6^ ^)^

Five-year survival rates of oral and oropharyngeal cancer are approximately 50%. The majority of patients live for a short time after diagnosis,^(^ ^7^ ^)^ because most tumors are identified late, compromising treatment, prognosis, and survival of patients.^(^ ^8^ ^,^ ^9^ ^)^ Therefore, it is necessary to disseminate information and statistical data regarding oral and oropharyngeal cancer to encourage professionals towards carrying out actions for early detection, contributing to a better understanding of the disease and of more feasible therapeutic proposals, and consequently increasing survival rates.^(^ ^10^ ^)^

## OBJECTIVE

To analyze the epidemiological profile and survival of patients with oral and oropharyngeal cancer at a university hospital.

## METHODS

This was a cross-sectional study by analysis of pathological reports of patients diagnosed with oral and oropharyngeal cancer at a university hospital in the Southern Region of the country, between January 2004 and December 2014. This is a teaching hospital integrated to a federal university in the city of Santa Maria (RS), which is considered a reference for the central region of the state.

The information collected was stored in the Teaching Information System, a digital system of the Department of Pathology of the university. In these reports, we selected the patients diagnosed with SCC of the mouth and oropharynx. The following variables were collected: municipality, sex, age, ethnicity, schooling level, histology grade, and anatomical site. Incomplete reports and patient data of those who had recurring cancer of the mouth and oropharynx were excluded.

Ethnicity was divided into white and non-white. Schooling level was divided into 8 years or less of education, and more than 8 years, and age was divided into decades (≤49, 50-59, 60-69, and ≥70 years).

The histologic grade was divided into three categories: well-differentiated, moderately differentiated, and poorly differentiated. Regarding anatomical site of tumor, the cases were considered according to codes of the International Classification of Diseases (ICD), tenth edition, in which C00 and C06 correspond to cancer of the mouth, and C10, to cancer of the oropharynx.

Data on mortality were acquired from the Mortality Information System (SIM) (NIS/DAT/CEVS/SES/RS - http://www2.datasus.gov.br/DATASUS/index.php?area=060701). For the survival analysis calculation, Kaplan-Meier method was used with information from the date of histopathological diagnosis until the date of death. After the descriptive analysis, the log-rank test was used to assess the factors related to survival. This analysis was done using Cox's regression model and p<0.05 was considered statistically significant. Data were analyzed using the Stata 12.0 software (Stata Corporation; College Station, Texas, United States).

The study was approved by the Ethics in Research with Human Beings Committee of the *Universidade Federal de Santa Maria*, opinion no. 924.661, CAAE protocol: 39197314.5.0000.5346.

## RESULTS

Of 254 patients diagnosed with oral and oropharyngeal cancer between 2004 and 2014, only 155 were included in the study since their data were complete in the reports analyzed. Among 155 patients, 39% were from the municipality of Santa Maria, and the other 61% were residents of nearby cities. As to sex, 87% were male and 13%, female. The most affected age group was the fifth decade of life, with a minimum age of 25 and a maximum of 86 years. As to race/ethnicity, most were white, corresponding to 95% of cases, and non-white to 5%. As to schooling level, 90% of individuals analyzed had less than 8 years of formal education.

The tongue was the most prevalent site (28%), followed by other parts of the mouth (23%), lips (20%), oropharynx (15%), and floor of the mouth (14%). As to distribution of cases according to histologic grade of the lesion, 49% presented with moderately differentiated SCC, 33% well-differentiated, and 12% poorly differentiated (Table 1).

**Table 1:** Characteristics of patients and tumors

Variables		n (%)
Municipality		
	Santa Maria	61 (39)
	Other cities	94 (61)
Sex		
	Male	135 (87)
	Female	20 (13)
Ethnicity		
	White	148 (95)
	Non-white	7 (5)
Schooling level, years		
	<8	139 (90)
	≥8 years	16 (10)
Age, years		
	≤40	37 (24)
	50-59	48 (31)
	60-69	43 (28)
	≥70	27 (17)
Anatomical site		
	Tongue	43 (28)
	Other parts of the mouth	34 (23)
	Lips	31 (20)
	Oropharynx	24 (15)
	Floor	23 (14)
Histological grade		
	Well differentiated	55 (36)
	Moderately differentiated	77 (49)
	Poorly differentiated	19 (12)
	Not specified	4 (3)

**Figure 1:**
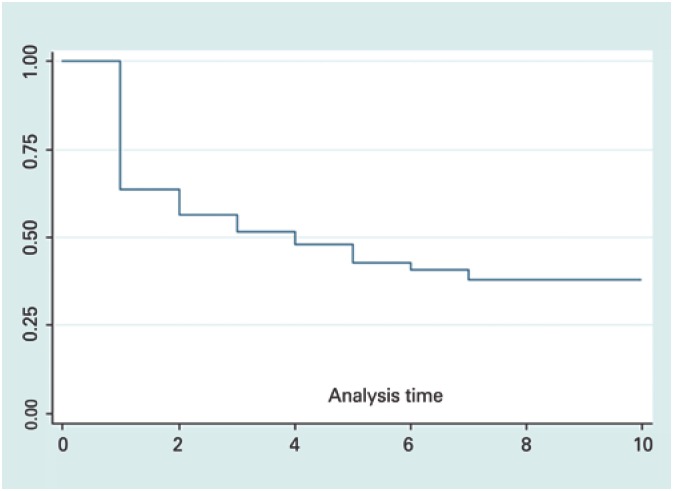
Kaplan-Meier survival analysis for oral and oropharyngeal cancer

**Figure 2:**
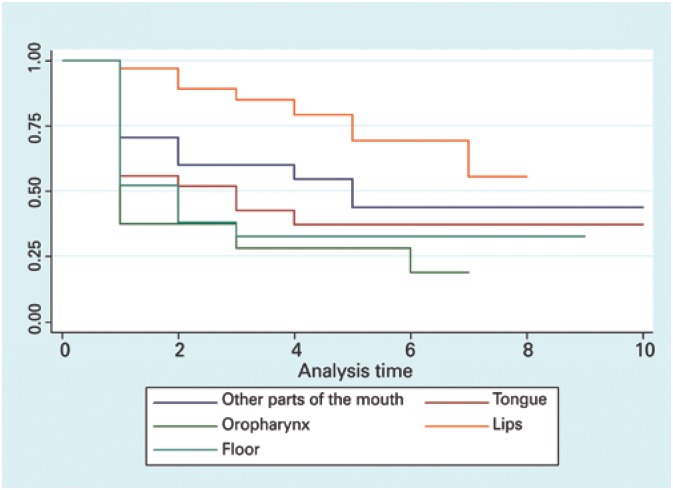
Kaplan-Meier survival analysis for site of oral and oropharyngeal cancer

As to death rates, 49% died due to oral and oropharyngeal cancer over the 10-year period. The mean survival time was 4 years (95% confidence interval − 95%CI 4.44-5.90). In 5 and 10 years, the survival rate was 42% and 38%, respectively. Overall survival is represented in figure 1. There were no statistically significant differences relative to survival according to age, sex, ethnicity, schooling, and histologic grade (Table 2). In reference to survival related to site, there was statistical difference (p=0.001), in which patients with cancer of the lip had a better survival rate (p=0.04), and patients with oropharyngeal cancer, the worst survival (p=0.03) (Figure 2).

**Table 2:** Oral and oropharyngeal cancer survival, according to patient and tumor variables

Variables		No death n (%)	Death n (%)	p value
Sex				
	Male	66 (49)	69 (51)	0.78
	Female	11 (55)	9 (45)	
Ethnicity				
	White	75 (51)	73 (49)	0.8
	Non white	3 (43)	4 (57)	
Schooling level, years				
	<8	65 (47)	74 (53)	
	≥8	11 (69)	5 (31)	0.08
Age, years				
	≤40	13 (35)	24 (65)	
	50-59	25 (52)	23 (48)	
	60-69	23 (53)	20 (47)	
	≥70	16 (59)	11 (41)	0.3
Anatomical site				
	Tongue	20 (47)	23 (53)	
	Other parts of the mouth	18 (53)	16 (47)	
	Lip	24 (77)	7 (23)	
	Oropharynx	7 (29)	17 (71)	
	Floor	8 (35)	15 (65)	0.001
Histological grade				
	Well differentiated	33 (59.9)	25 (43.1)	
	Moderately differentiated	44 (55)	36 (45)	
	Poorly differentiated	11 (55)	9 (45)	0.54
	Not specified	44 (51.16)	42 (48.84)	

p value: log-rank test.

## DISCUSSION

Cancer of the mouth and oropharynx is characterized by high prevalence, mortality, and low survival rates.^(^ ^11^ ^)^ In this study, we evaluated the epidemiological profile and the survival rate of patients with oral and oropharyngeal cancer, diagnosed at a hospital in the countryside of the State of Rio Grande do Sul. Results showed a low survival rate in these individuals. Additionally, the anatomical tumor site showed a significant association with survival, since individuals with cancer of the lip presented with better percentages, while those whose disease was in the oropharynx had the worst rates.

In this study, the majority of patients who sought treatment at the hospital were from neighboring cities, highlighting the importance of these services and of reference hospitals to treat the population. In general, the epidemiological profile of these patients was similar to other studies in the literature.^(^ ^12^ ^,^ ^13^ ^)^ Men were more often diagnosed with oral cancer than women, probably for being more exposed to risk factors. However, the number of women affected by this neoplasm has been growing over the years, since they are now exposing themselves more to tobacco and alcohol.^(^ ^14^ ^)^ Also identified in this research was a higher frequency of cancer in white individuals and in the fifth decade of life, as well as the higher prevalence at the site of the tongue and moderate histologic grade, which are characteristics also described in other studies.^(^ ^15^ ^–^ ^17^ ^)^

As to survival, in this study, we noted a low rate, corresponding 5 and 10 years, respectively, to 42% and 38%. These results are better than those found the Southern region of Thailand, where the 5 and 10 year survival were 24.1% and 25.95%, respectively. The authors attributed these findings to the advanced stage of the disease when patients were diagnosed, and to the type of treatment provided.^(^ ^18^ ^)^ Nevertheless, a study carried out in the Netherlands, from 1989 to 2011, demonstrated patients diagnosed with oral and oropharyngeal SCC responded better to treatments, increasing the survival rate to 67% and 48%, respectively in oral cavity and oropharyngeal cancers.^(^ ^19^ ^)^ Therefore, we note that survival rates are better in more developed countries as compared to developing ones.^(^ ^8^ ^)^

The present study demonstrated the variables age, sex, ethnicity and schooling did not show a significant association with survival rates of oral and oropharyngeal cancer, just as in the study by Schneider et al.^(^ ^20^ ^)^ However, in an investigation conducted in São Paulo from 1999 to 2002, the authors observed that individuals with more advanced age presented with the worst survival rates, and this, as per the authors, could be related to the occurrence of debilitating diseases and other complications associated with aging.^(^ ^21^ ^)^ A recent study aimed to evaluate the influence of ethnicity on survival of oropharyngeal cancer patients, and the variable showed a significant association, unlike the present study. The authors noted that black patients had the worst survival rates, which were probably attributed to the worst socioeconomic conditions of these individuals, hindering access to treatment.^(^ ^22^ ^)^

It was also noted that the histologic grade showed no significant relation to survival of patients with oral and oropharyngeal cancer. Nevertheless, Kademani et al.,^(^ ^23^ ^)^ demonstrated the histologic grade was a predictive factor for oral cancer, and the tumors presenting with a poorly differentiated grade had the worst survival rates. According to the authors, this finding was justified by the fact that neoplasms with this histologic characteristic presented with a greater prevalence of cervical metastasis. Here, the histologic grade may not have shown an association with the survival rates due to limitations of the study design − since the retrospective studies evaluate the patients’ medical records, which contained incomplete data, thus diminishing the sample in the present investigation.

As to the anatomical site of the tumor, patients with cancer in the lip region had better survival rates, and those located in the oropharynx had the worst rates. Sites with more blood and lymph vessels, besides difficult to assess sites that hinder the diagnosis and treatment, can influence the progression and prognosis of the tumor.^(^ ^24^ ^)^ In this regard, the lip region is more accessible, facilitating early detection and diagnosis, resulting, consequently, in better survival rates.^(^ ^25^ ^)^

Oropharyngeal cancer is strongly associated with cervical metastases, with incidence of 50 to 70%,^(^ ^26^ ^)^ due to its greater tumor dissemination, besides being located in regions difficult to visualize and diagnose, contributing negatively to patient survival,^(^ ^27^ ^)^ as was observed in this study. Additionally, HPV-positive patients diagnosed with oropharyngeal cancer are related to a better prognosis than those who are HPV-negative. This fact can be related to the low percentage of mutation present in these tumors, predisposing towards better responses to treatment and high survival rates,^(^ ^28^ ^)^ differently from that found in this study. Nevertheless, in the present study, it was not possible to evaluate this association due to the absence of data in the system on presence of HPV. Further studies should be conducted to evaluate the influence of HPV in oropharyngeal cancers.

## CONCLUSION

The survival rate of patients with oral and oropharyngeal cancer was shown to be low in this study. The anatomical site influenced patient survival, in which tumors located in the oropharynx presented with worse survival rates, while those located on the lip had the best rates.
